# Body mass index, blood glucose, and mortality in patients with ischemic stroke in the intensive care unit: A retrospective cohort study

**DOI:** 10.3389/fnins.2022.946397

**Published:** 2022-10-19

**Authors:** Zisheng Ma, Shunxian Li, Xinjiang Lin

**Affiliations:** Department of Neurology, The First Affiliated Hospital of Shantou University Medical College, Shantou, Guangdong, China

**Keywords:** body mass index, glucose, mortality, ischemic stroke, intensive care unit

## Abstract

**Background:**

Excessive BMI was associated with lower mortality after stroke. However, some believed that excessive BMI can lead to a poor prognosis because of some physiological mechanism, such as glucose metabolism disorder. Therefore, this study aims to discuss the association between mortality, BMI, and blood glucose.

**Materials and methods:**

This was a retrospective observational study and all data were extracted from the Medical Information Mart for Intensive Care III database. The exposure was BMI classified into the normal weight group and the excessive weight group. The outcome concluded 30-day, 90-day, and 1-year mortality. The association between two groups and mortality was elucidated by Cox regression models, propensity score matching (PSM) and inverse probability of treatment weighting (IPTW). The underlying effect of blood glucose on the “obesity paradox” was analyzed by causal mediation analysis.

**Results:**

According to Cox regression models, a significant beneficial effect of excessive BMI in terms of mortality was observed: 30-day mortality (HR 0.57, 95% CI 0.35–0.90, *P* = 0.017), 90-day mortality (HR 0.53, 95% CI 0.36–0.78, *P* = 0.001), and 1-year mortality (HR 0.65, 95% CI 0.46–0.91, *P* = 0.013). After PSM and IPTW, we got a similar conclusion. The causal mediation analysis showed that the protective effect of excessive BMI on 30-day mortality reduced with the increase of blood glucose.

**Conclusion:**

For ischemic stroke patients in the Intensive Care Unit, those with excessive BMI are associated with both lower short-term mortality and lower long-term mortality, while the protective effect on 30-day mortality weakened accompanied by the increase of blood glucose.

## Introduction

Stroke is a multifactorial and global pandemic disease, with the second leading cause of death and the third leading cause of disability, which imposes enormous medical, economic, and social burdens (Campbell and Khatri, 2020). As reported, the three most dominant risk factors for stroke are hypertension, high body mass index, and high blood glucose, in which the high BMI is the fastest-growing risk factor (Feigin et al., 2021). Although excessive BMI were associated with the higher morbidity, it can also link to the lower mortality after onset, which is called obesity paradox. Obesity paradox has been observed in atrial fibrillation (Proietti et al., 2017), heart failure (Oreopoulos et al., 2008), coronary artery disease (Uretsky et al., 2007), acute myocardial infarction (Colombo et al., 2015), end-stage kidney (Ahmadi et al., 2015).

In stroke patients, the obesity paradox had been demonstrated in a recent review that included 25 studies with about 300,000 cases (Bagheri et al., 2015). But there are still some articles holding different opinions (Ryu et al., 2011; Dehlendorff et al., 2014). All previous articles discussed the protective effect of excessive BMI in the total stroke population, but lack of research for critically ill stroke patients. However, critically ill patients tend to be at greater risk of death and need to be handled more carefully. Besides, in the clinical work of the intensive care unit (ICU), a normal weight is often not considered as a risk factor and cannot attract special attention. Therefore, it is meaningful to conduct a separate analysis to directedly show the relationship between excessive BMI and mortality for ischemic stroke patients in the ICU.

Evidence suggested that hyperglycemia was not only an independent risk factor for stroke but also contributed to a worse prognosis in stroke patients regardless of diabetes (Masrur et al., 2015; Zhu et al., 2017). Unfortunately, when the body suffers from an acute illness, it can cause a transient increase in blood glucose, even in those without glucose metabolism disorder before, which is known as stress hyperglycemia. Besides, insulin resistance was proposed to be one of the causes of stress hyperglycemia (Ertorer et al., 2010). Due to this, obesity, associated with insulin resistance and glucose tolerance (Borschmann et al., 2017; Larsson et al., 2017), may aggravate acute blood glucose fluctuations and thus lead to a worse outcome in critically ill patients with stroke. This contradicts the obesity paradox. These findings led us to question whether the protective effect of high BMI on prognosis will be masked or weakened by hyperglycemia. Therefore, we designed this study to talk about the obesity paradox in critically ill patients and the potential role of blood glucose in this process.

## Materials and methods

### Study population

The present subject is a retrospective cohort based on the data stored in the MIMIC III version v1.4 (Medical Information Mart for Intensive Care). Briefly, the MIMIC-III database is a large freely accessible critical single-center database and contains more than 50,000 ICU admissions between June 2001 and October 2012 (Johnson et al., 2016). In the present analysis, 943 patients with ischemic stroke in the MIMIC III database were extracted according to the International Classification of Diseases (ICD-9) codes. The participants with ischemic stroke in the ICU (MIMIC III database) were defined as critically ill or severe stoke patients in the current study. Finally, we involved 522 patients, while 421 of them were excluded according to the following criteria: (1) Non-first ICU stays; (2) Died within the first 24 h; (3) Lack of height and weight data; (4) Without complete data of covariates; (5) BMI less than 18.5. There were only 14 patients with BMI less than 18.5, it was too minimal to draw a significant conclusion. The patients with BMI less than 25 were defined as the normal weight group, while else were defined as the excessive weight group. We also conducted a comparison of baseline characteristics between included and excluded patients and between included and all patients to discuss the selection bias.

### Data extraction

The present study was conducted in the ICU. The first day following the ICU admission was defined as the first day in the analysis. Baseline characteristics were collected using structured query language, including age, gender, weight, height, date of birth and death, and comorbidities (hypertension and diabetes). The vital signs and physiology variables within the first day were also recorded, including systolic pressure, diastolic pressure, heart rate, respiratory rate, temperature, blood urea nitrogen, and creatinine. We use the average of each variable if they were measured more than once. The blood glucose concentration in the acute period was defined as the average of all random blood glucose measurements within the first 24 h.

In stroke patients, it is widely accepted that the NIH stroke score (NHISS) was a specific scale to evaluate the severity of illness and predict prognosis. Unfortunately, in the ICU, patients with stroke always often lack accurate NHISS on admission due to several reasons, such as coma, unstable vital signs, and poor mental status. In addition, stroke patients are often complicated with multiple system injuries and face a complex risk of death. Therefore, in the present study, critical illness scores involving multiple systems at admission were adopted to assess the disease severity, for example, Sequential Organ Failure Assessment (SOFA), Simplified Acute Physiology Score II (SAPS II), and Elixhauser Comorbidity Index (ECI) score (Le Gall et al., 1984; Vincent et al., 1996; van Walraven et al., 2009). The SOFA score is a prediction system for assessing the incidence of organ dysfunction or failure in ICUs. SAPS II score includs 12 physiological variables, age, type of hospitalization, and 3 chronic diseases. It is reported that a higher SAPS II score means worse disease severity and worse outcome. ECI score is a comprehensive scoring system for evaluating the prognosis of patients in the ICU by baseline diseases and has an advantage in predicting long-term outcomes.

### Statistical analysis

The skewness distribution was presented as median (quartile) while the categorical variables were presented as total number (percentage). The baseline characteristics of different groups were analyzed by the Mann–Whitney *U* test (continuous variables) and the χ^2^ test or Fisher’s exact test (categorical variables). The effect of BMI on survival was investigated by the Cox regression models. To assess confounding, those related to the outcome in the univariate analysis (*p* < 0.10) were entered into a Cox regression model in the basic model or eliminated the covariates in the complete model one by one, then compared the regression coefficients. Those covariates altering initial regression coefficients by more than 10% were adjusted in the multivariate Cox regression model. The interactions between BMI and sex were analyzed in the subgroup analysis.

We used propensity score matching (PSM) to reduce the potential confounding between the normal group and the excessive group. A multivariable logistic regression model was performed to compute the propensity score, including crucial variables which are known to impact clinical stroke outcomes showing in Table 1: age, gender, comorbidity, vital signs, SOFA, SAPS II, ECI, and blood glucose. For PSM, we adopted the one-to-one nearest neighbor matching approach with a caliper size of 0.2. We also created an inverse probability of treatment weighting (IPTW) to adjust for confounding due to differences between the groups. The weight for the excessive group was defined as pt/ps, while the weight for the normal group was defined as (1-pt)/(1-ps). The detailed of IPTW did not show in the present article. Standardized mean difference (SMD) was adopted to evaluate the balance between covariates. A SMD of less than 10% for a baseline covariate reveals a negligible imbalance.

**TABLE 1:** Characteristics of the study population stratified into two classifications of body mass index (BMI).

Variables	Original cohort				Matched cohort		
	Normal weight	Excessive weight	*p*	SMD	Normal weight	Excessive weight	SMD
*N*	177	345			166	166	
Age, years	76.0 (64.0, 83.0)	67.0 (57.0, 77.0)	**<0.001**	0.338	75 (63.0, 82.0)	72 (64.0, 80.0)	0.038
Male, *n* (%)	75 (42.4%)	182 (52.8%)	**0.031**	0.209	72 (43.9%)	68 (41.0%)	0.049
Comorbidity							
hypertension	29 (16.4%)	51 (14.8%)	0.724	0.044	27 (16.3%)	26 (15.7%)	0.016
diabetes	41 (23.2%)	129 (37.4%)	**0.001**	0.313	41 (24.7%)	43 (25.9%)	0.028
heart failure	35 (19.8%)	64 (18.6%)	0.826	0.031	33 (18.1%)	27 (16.3%)	0.048
cardiac arrhythmias	69 (39.0%)	116 (33.6%)	0.265	0.112	65 (39.2%)	65 (38.0%)	0.025
chronic pulmonary	41 (23.2%)	63 (18.3%)	0.226	0.121	36 (21.7%)	36 (21.7%)	<0.001
renal failure	32 (18.1%)	56 (16.2%)	0.682	0.049	30 (18.1%)	31 (18.7%)	0.016
liver disease	7 (4.0%)	14 (4.1%)	1	0.005	6 (3.6%)	8 (4.8%)	0.06
Vital signs							
SBP, (mmHg)	125.1 (113.4, 142.8)	130.0 (115.6, 143.9)	0.148	0.129	126.0 (115.4, 143.7)	126.0 (115.4, 143.7)	0.032
DBP, (mmHg)	62.4 (56.0, 67.7)	63.8 (55.6, 73.2)	0.083	0.198	62.6 (56.2, 68.5)	61.7 (54.5, 71.5)	0.059
Heart rate, (bpm)	81.0 (68.5, 95.3)	80.7 (71.3, 91.6)	0.83	0.038	79.8 (68.4, 94.5)	80.7 (71.5, 94.5)	0.004
Respiratory rate, (bpm)	18.3 (16.4, 21.0)	18.4 (16.3, 20.5)	0.937	0.006	18.1 (16.3, 21.0)	18.6 (16.2, 21.2)	0.051
Temperature, (°C)	36.8 (36.5, 37.2)	36.8 (36.5, 37.2)	0.918	0.037	36.8 (36.5, 37.2)	36.8 (36.5, 37.2)	0.033
Score							
SAPS II	37.0 (29.0, 45.0)	31.0 (26.0, 42.0)	0.002	0.238	37.0 (28.0, 45.0)	34.0 (27.0, 45.0)	0.04
SOFA	3.0 (2.0, 6.0)	3.0 (2.0, 5.0)	0.731	0.011	3.0 (2.0, 6.0)	3.0 (2.0, 5.0)	0.098
ECI	5.0 (0.0, 12.0)	5.0 (0.0, 11.0)	0.088	0.136	5.0 (0.0, 12.0)	5.0 (0.0, 11.0)	0.061
Glucose, (mg/dL)	123.7 (107.5, 143.3)	133.0 (116.8, 158.7)	<0.001	0.339	123.9 (108.2, 144.9)	128.0 (113.5, 147.9)	0.041

SBP, systolic blood pressure; DBP, diastolic blood pressure; SOFA, sequential organ failure assessment; SAPS II, simplified acute physiology score II; ECI, Elixhauser-vanwalraven comorbidity index.

Bold values indicate that they are statistically significant.

To explore the potential effect of blood glucose on the obesity paradox, we conducted the causal mediation analysis by the “mediation” R package (Zhang et al., 2016). The analysis was conducted by the Bootstrap test and the seeds were set to 2,000. The results would be presented as average causal mediation effect (ACME), average direct effect (ADE), and total effect.

All the analyses were performed with the statistical software packages R 3.3.2 (The R Foundation).^1^ A two-tailed test was performed and *p* < 0.05 was considered statistically significant. *P*-values less than 0.05 (two-sided) were considered statistically significant.

## Results

### Characteristics and survival outcomes

In the original cohort, 522 patients who fulfilled the inclusion criteria were admitted to the analysis and were divided into two groups by BMI: a normal weight group (BMI < 25, *n* = 177) and excessive weight group (BMI ≥ 25, *n* = 345) (Table 1). No selection bias was found between included and excluded patients or between included and all patients. Compared with the normal weight group, the group with excessive BMI was more likely to be male (52.8 vs. 42.4%, *p* = 0.031) or younger (median: 67 vs. 76, *p* < 0.001). The excessive weight group had a lower proportion of diabetes, but higher admission blood glucose levels. In the present study, excessive weight patients had lower SAPS II scores (Median: 31.0 vs. 37.0, *P* = 0.002), but there was no significant difference in SOFA (Median: 3.0 vs. 3.0, *P* = 0.731) and ECI scores (Median: 5.0 vs. 5.0, *P* = 0.081). After PSM and IPTW, all variables showed no statistical difference (SMD < 0.1) (Table 1). We found that, in critically ill patients with ischemia stroke, the excessive weight group had lower mortality at 30 and 90 days, and at 1 year (Table 2).

**TABLE 2:** Outcomes of normal weight group and excessive weight group.

Mortality	Original cohort			Matched cohort		
	Normal group	Excessive group	*p*	Normal group	Excessive group	*p*
30-day, *n* (%)	39 (22.0%)	46 (11.6%)	0.002	35 (21.1%)	17 (10.2%)	0.01
90-day, *n* (%)	56 (31.6%)	54 (15.7%)	<0.001	52 (31.3%)	22 (13.3%)	<0.001
1-year, *n* (%)	66 (37.3%)	79 (22.9%)	<0.001	62 (37.3%)	32 (19.3%)	<0.001

### Body mass index and mortality

Univariate analysis showed that the dominant factors for 30-day mortality were age, heart rate, SOFA, SAPS II, and ECI scores and glucose, while the dominant factors for 90-day and 1-year mortality were age, heart rate, SOFA, SAPS II, and ECI scores (Table 3). To assess confounding, covariates including age, heart rate, respirate rate, SOFA, SAPS II, ECI, and glucose were entered into a Cox regression model in the basic model, or were eliminated one by one in the complete model, then the regression coefficients were compared. Initial regression coefficients altering by more than 10% were considered to be significant. Therefore, we adjusted the gender, age, SAPS II score, and glucose when analyzing the association between BMI and 30-day mortality by the multivariate Cox regression analysis (Table 4). In addition, we adjusted the covariates, including gender, age, and SAPS II in the Cox regression analysis for 90-day and 1-year mortality. Results indicated that excessive BMI was related to lower 30-day (HR 0.57, 95% CI 0.35–0.90, *p* = 0.017), 90-day (HR 0.53, 95% CI 0.36–0.78, *p* = 0.001), and 1-year (HR 0.65, 95% CI 0.46–0.91, *p* = 0.013) mortality. After PSM and IPTW, we obtained a similar conclusion for 30-day mortality (PSM: HR 0.46, 95% CI 0.26–0.82, *p* = 0.008; IPTW: HR 0.56, 95% CI 0.36–0.87, *p* = 0.001), 90-day mortality (PSM: HR 0.38, 95% CI 0.23–0.63, *p* < 0.001; IPTW: HR 0.53, 95% CI 0.36–0.76, *p* < 0.001), and 1-year mortality (PSM: HR 0.45, 95% CI 0.30–0.69, *p* < 0.001; IPTW: HR 0.66, 95% CI 0.48–0.91, *p* = 0.013) (Table 4). The Subgroup analyses for the relationship between BMI and mortality were performed by sex. As shown in Table 5, the conclusion was stable in all subgroups (*P* for interaction > 0.05).

**TABLE 3:** Dominant mortality factors by univariable analysis.

Variables	30-day mortality		90-day mortality		1-year mortality	
	HR (95% CI)	*p*	HR (95% CI)	*p*	HR (95% CI)	*p*
Excessive BMI	0.49 (0.32,0.77)	**0.002**	0.45 (0.31,0.66)	**<0.001**	0.55 (0.39,0.72)	**<0.001**
Male	0.94 (0.61,1.47)	0.793	0.95 (0.65,1.38)	0.777	0.9 (0.65,1.24)	0.506
Age	1.01 (1.00,1.01)	**<0.001**	1.00 (1.00,1.01)	**<0.001**	1.00 (1.00,1.01)	**<0.001**
Hypertension	0.69 (0.35,1.39)	0.303	1.05 (0.63,1.74)	0.854	1.25 (0.82,1.9)	0.297
Diabetes	0.89 (0.55,1.44)	0.637	1.00 (0.67,1.49)	0.987	1.05 (0.75,1.48)	0.775
Heart rate	1.02 (1.00,1.03)	**0.019**	1.02 (1.01,1.03)	**0.004**	1.01 (1,1.02)	**0.009**
Systolic pressure	1.00 (0.99,1.01)	0.771	1.00 (0.99,1.01)	0.364	0.99 (0.99,1.00)	0.166
Diastolic pressure	0.99 (0.97,1.01)	0.155	0.99 (0.97,1.00)	0.17	0.99 (0.98,1.00)	0.134
Respiratory rate	1.08 (1.03,1.13)	**0.002**	1.06 (1.01,1.10)	**0.016**	1.05 (1.01,1.09)	**0.024**
Temperature	1.29 (0.89,1.88)	0.177	1.22 (0.89,1.67)	0.223	1.10 (0.83,1.45)	0.513
SOFA	1.10 (1.03,1.17)	**0.006**	1.1 (1.04,1.17)	**<0.001**	1.08 (1.03,1.14)	**0.002**
SAPS II	1.04 (1.03,1.06)	**<0.001**	1.04 (1.03,1.05)	**<0.001**	1.04 (1.03,1.05)	**<0.001**
ECI	0.69 (0.35,1.39)	**0.004**	1.04 (1.02,1.06)	**<0.001**	1.05 (1.03,1.07)	**<0.001**
Glucose	1.01 (1.00,1.01)	**0.033**	1.00 (1.00,1.01)	0.253	1.00 (1.00,1.00)	0.749

SOFA, Sequential Organ Failure Assessment; SAPS II, Simplified Acute Physiology Score II; ECI, Elixhauser-vanwalraven Comorbidity Index.

Bold values indicate that they are statistically significant.

**TABLE 4:** Association between the excessive body mass index (BMI) and mortality in critically ill patients with acute ischemic stroke.

Variables	30-day mortality		90-day mortality		1-year mortality	
	HR (95% CI)	*p*	HR (95% CI)	*p*	HR (95% CI)	*p*
Multivariate Cox regression	0.57 (0.35, 0.90)^a^	0.017	0.53 (0.36, 0.78)^b^	0.001	0.65 (0.46, 0.91)^b^	0.013
PSM	0.46 (0.26, 0.82)	0.008	0.38 (0.23, 0.63)	<0.001	0.45 (0.30, 0.69)	<0.001
IPTW	0.56 (0.36, 0.87)	0.001	0.53 (0.36, 0.76)	<0.001	0.66 (0.48, 0.91)	0.013

^a^, adjusted for gender, age, SAPS II, glucose; ^b^, adjusted for gender, age, SAPS II; PSM, propensity score matching; IPTW, inverse probability of treatment weighting.

**TABLE 5:** Association between body mass index (BMI) and ischemic stroke outcomes stratified by age, sex, and diabetes.

Variables	30-day mortality		90-day mortality		1-year mortality	
	HR (95% CI)	*P* for interaction	HR (95% CI)	*P* for interaction	HR (95% CI)	*P* for interaction
Gender		0.118		0.059		0.087
Male (257)	0.38 (0.19 0.74)		0.35 (0.20 0.61)		0.46 (0.28 0.75)	
Female (265)	0.81 (0.43 1.55)		0.77 (0.45 1.31)		0.87 (0.55 1.38)	

### Causal mediation analysis

The excessive weight group has higher level of blood glucose concentration (Median: 123.7.0 vs. 133.0, *P* < 0.001) (Table 1). Excessive BMI is associated with a lower 30-day mortality (Table 4), while the blood glucose concentration is associated with higher 30-day mortality (Table 3). To validate how BMI affects mortality, we next performed causal mediation analysis on the causal structure (Table 6). The ACME (indirect effect) was 0.019 (95% Cl, 0.004, 0.040; *p* = 0.008), the ADE (direct effect) was −0.181 (95% Cl, −0.309, −0.060; *p* = 0.001), and the total effect was −0.162 (95% Cl, −0.289, −0.040; *p* = 0.001). Although the excessive BMI group performed better on the 30-day mortality after stroke, it had a negative effect by enhancing the level of blood glucose, which reduced the protective effect of BMI on 30-day mortality by 11.94%.

**TABLE 6:** Causal mediation analysis for the effect of elevation of blood glucose on 30-day mortality.

	Estimate (95% CI)	*p*
ACME	0.019 (0.004, 0.040)	0.008
ADE	−0.181 (−0.309, −0.060)	0.001
Total effect	−0.162 (−0.289, −0.040)	0.001
Prop. Mediated	−11.94% (−3.00%, −42.35%)	0.009

ACME, average causal mediation effects (indirect effect); ADE, average direct effects (direct effect); Prop. Mediated: Conceptually ACME/Total effect (The proportion of mediating variables that mediate the outcome).

## Discussion

In our study, we confirmed in critically ill stroke patients that excessive BMI can decrease mortality, including short-term and long-term mortality. This finding was consistent with many previous articles (Bagheri et al., 2015), but there are still some opposite voices (Bagheri et al., 2015). Ryu et al. (2011) pointed out that when talking about the obesity paradox, the initial neurological severity may be a “mediator.” This is because there are clear differences in stroke patterns between patients with normal and excessive BMI, which lead to different degrees of initial neurological injury. It has been demonstrated that the main etiology of stroke in patients with low BMI is cardiac embolism, while the main etiology of stroke in patients with high BMI is small vessel occlusion. Patients with high BMI are considered to have a higher proportion of lacunar infarction compared to atherosclerotic thrombosis and myocardial infarction (Tanizaki et al., 2000). This suggests that the protective effect of high BMI may be an illusion caused by lighter initial severity. In the present study, we use the SOFA, SAPS II, and ECI scores to assess the severity of the disease and the excessive weight group had a lower SAPS II score. In addition, univariable analysis showed that the SAPS II score was associated with higher mortality. Therefore, we further adjusted the impact of admission SAPS II scores in the multivariate Cox regression analysis and found that the correlation between BMI and mortality rate was reduced, but still significant.

In subgroup analysis, there was no interaction between BMI and sex. Contrarily, a reported article claimed that, in ischemic stroke patients, the mortality benefits associated with obesity are restricted in men, while the same conclusion could not be reached in women (Saini et al., 2014). But differ from our study, the previous study did not discuss the interaction between BMI and sex in their work. There are a few theories supporting sex difference between males and females. First, BMI is calculated by height and weight and cannot directly show the percentage of lipid variation across sexes. In general, thought with the same body mass index, women generally have a higher percentage of body fat than men. Second, women store more lipid in the gluteal-femoral region, whereas men store more in the visceral depot (Blaak, 2001; Bagheri et al., 2015), which often leads to a better outcome in females (Strulov Shachar and Williams, 2017; Cao et al., 2018). Third, sex hormone differences may underlie the observed gender disparity (WHO Expert Consultation, 2004). However, in our study, the protective effect of excessive BMI was not modified by sex.

The reason for the “obesity paradox” is not evident at present and several hypotheses regarding the role of obesity in stroke pathogenesis have been proposed. First, the increased nutritional reserves provided by excess fat stores may provide an added advantage in the first moments of acute illness (Gioulbasanis et al., 2011; Karagozian et al., 2016). Second, as documented by several studies (Wannamethee and Atkins, 2015; Ebadi et al., 2017), the obesity paradox can emerge because patients with higher weight often have higher levels of protective muscle mass. Third, it has been reported that a stronger ability to resist inflammation (Haley and Lawrence, 2016; Rodríguez-Castro et al., 2019), and oxidative stress (Salie et al., 2014) may be responsible for the obesity paradox. All in all, the exact mechanisms for obesity paradox remain controversial.

Stress hyperglycemia is common in acute stroke patients (Vancheri et al., 2005; Mitchell et al., 2012). A prospective observational study showed that about 60% of patients suffer from stress hyperglycemia (Dave et al., 2010). Patients with excessive BMI, are more likely to suffer hyperglycemia in the acute period, which is a strong negative prognostic indicator of death after stroke. Studies have demonstrated that increased fat mass can provoke disorders of glucose metabolism by an array of pathways (Song et al., 2006; King, 2008; Stienstra et al., 2010). In another experimental study, it is also reported that a high-fat diet could induce hyperglycemia and aggravate the growth of cerebral infarct volume (Grisotto et al., 2021). In our research, we found that the excessive BMI group exactly had a higher blood glucose concentration in the first 24 h, and the level of blood glucose was also independently linked to lower 30-day mortality, but irrelevant to 90-day and 1-year mortality. Causal mediation analysis showed that excessive BMI is associated with lower mortality in total while worsening the 30-day prognosis by raising the level of blood glucose. This implies that BMI may have a two-sided effect on the mortality of stroke patients in the ICU.

As reported before, hyperglycemia is related to the growth of acute cerebral infarct volume, the disruption of the blood-brain barrier and the aggravation of brain edema after stroke, and often leads to a poorer outcome. In a study published in 2014, the glucose level on admission was shown to be associated with the growth of infarct volume (Shimoyama et al., 2014). Shimoyama et al. (2016) prospectively measured the serum glucose concentration every 5 min for up to 72 h and showed a significant correlation between hyperglycemia and infarct volume growth in patients with ischemic stroke. Moreover, research in mice has demonstrated that rats with hyperglycemia suffer from increased blood-brain barrier disruption and cerebral edema (Kamada et al., 2007; Huang et al., 2013; Desilles et al., 2017). The activation of inflammations and oxidative stress is thought to be the key mechanisms for the pathological changes mentioned above (Shukla et al., 2017). Contrarily, patients with excessive BMI have been proposed to more strongly resist inflammation (Rodríguez-Castro et al., 2019) and oxidative stress (Salie et al., 2014) after stroke. Perhaps, the different effects on inflammation and oxidative stress may be the cause for the opposite mediating role of glucose in the obesity paradox. To our knowledge, there is no preclinical study that can directly support this hypothesis and further study are need. A preclinical study observed a better performance of the diaphragm diaphragmatic function in obese rats, but not in diabetic obese rats (De Jong et al., 2017). The article also suggested that this difference was partly caused by the different activation of AKT pathway signaling. This inspired us that glucose may influence the benefit of obesity in ischemic stroke by specific pathway and further study are needed.

In our study, the obesity paradox is evident in critically ill patients with acute ischemic stroke. Also, we are the first ones to point out that excessive BMI may have negative implications on the prognosis of severe stroke patients by enhancing blood glucose levels. However, our study also has some limitations that need to be tackled in future studies. Firstly, since the data in MIMIC-III is generated within a single electronic medical record system, it might contain systematic biases. This may not allow to generalize the findings to other care settings, thus, more repetitive studies are needed to test the universality of our conclusion. Secondly, this is a retrospective study without follow-up data about weight change, home care and treatment schemes after discharge. Thirdly, though BMI is a convenient clinical index, it cannot provide accurate measurement of body fat content and distribution. So, some variables will become more significant, for example, waist circumference, waist-hip ratio, skinfold estimates, and bioelectrical impedance analysis. Fourthly, the underlying mechanism of our study remained unclear. Further preclinical work is need to better understand how excessive BMI and glucose affect the prognosis of stroke and how glucose decrease the mortality benefit associated with excessive BMI. The disclosure of mechanism may provide a new treatment strategy in the future.

## Conclusion

Excessive BMI is associated with lower mortality after severe stroke. However, excessive BMI plays a dual role in the short-term, 30-day prognosis of stroke patients in the ICU. Not only can it reduce the risk of death, but also can worsen the outcome by increasing the levels of blood glucose.

## Data availability statement

The datasets presented in this article are not readily available because as the data cannot be made publicly available due to the regulation of the database MIMIC-III database. Researchers should apply their own study plans to the website of MIMIC database. After finishing the ethical review, a username and password will be provided for them to access the complete data at: https://archive.physionet.org/works/MIMICIIIClinicalDatabase/files/.

## Author contributions

ZM: conceptualization, data curation, formal analysis, methodology, software, visualization, and writing—original draft preparation. SL: formal analysis, methodology, validation, and writing—original draft preparation. XL: conceptualization, methodology, project administration, supervision, and writing—review and editing. All authors contributed to the article and approved the submitted version.

## Footnotes

1
http://www.R-project.org
